# Involvement of MrgprC in Electroacupuncture Analgesia for Attenuating CFA-Induced Thermal Hyperalgesia by Suppressing the TRPV1 Pathway

**DOI:** 10.1155/2018/9102107

**Published:** 2018-02-12

**Authors:** Ying-jun Liu, Xiao-xi Lin, Jian-qiao Fang, Fang Fang

**Affiliations:** Department of Neurobiology and Acupuncture Research, The Third Clinical Medical College, Zhejiang Chinese Medical University, Hangzhou 310053, China

## Abstract

Mas-related G-protein-coupled receptor C (MrgprC) plays an important role in modulating chronic inflammatory pain. Electroacupuncture (EA) has a satisfactory analgesic effect on chronic pain. This study aimed to investigate the involvement of MrgprC and its transient receptor potential vanilloid 1 (TRPV1) pathway in EA analgesia in chronic inflammatory pain. Chronic inflammatory pain was induced by subcutaneously injecting complete Freund's adjuvant (CFA) into the left hind paw. EA (2/100 Hz) stimulation was administered. MrgprC siRNAs were intrathecally administered to inhibit MrgprC expression, and bovine adrenal medulla 8-22 (BAM8-22) was used to activate MrgprC. The mechanical allodynia was decreased by EA significantly since day 3. The piled analgesic effect of EA was partially blocked by 6 intrathecal administrations of MrgprC siRNA. Both EA and BAM8-22 could downregulate the expression of TRPV1 and PKC in both the DRG and the SCDH. Both EA and BAM8-22 could also decrease the TRPV1 translocation and p-TRPV1 level in both the DRG and the SCDH. The effects of EA on PKC*ε*, TRPV1 translocation, and p-TRPV1 in both the DRG and the SCDH were reversed by MrgprC siRNA. The results indicated that MrgprC played crucial roles in chronic pain modulation and was involved in EA analgesia partially through the regulation of TRPV1 function at the DRG and SCDH levels.

## 1. Introduction

Chronic inflammatory pain is a kind of refractory disease, and the prevalence rate in developing countries is higher than developed countries, with approximately 33% in the general adult population and 56% in the elderly population [1]. Cyclooxygenase-2 (COX-2), tumor necrosis factor-*α* (TNF-*α*), substance P (SP), and other neurotransmitters that are stimulated by sustained inflammation activate ion channels such as transient receptor potential vanilloid 1 (TRPV1) and acid-sensing ion channels (ASICs) and then induce peripheral pain sensitization [2]. The ongoing input conveyed from peripheral neurons overexcites spinal nociceptive neurons, and finally central sensitization is induced. On the other hand, nociceptive information transferred from the periphery to the spinal cord can also be modulated in peripheral sensory neurons, especially in the dorsal root ganglia (DRG), through the activation of the opioid system [3, 4].

Electroacupuncture (EA), a commonly used acupuncture method, has been widely promoted for alleviating chronic inflammatory pain [5]. Previous studies of EA analgesia mainly focused on spinal and supraspinal mechanisms. However, we found that local acupuncture can achieve better analgesic effects than distal acupuncture for treatment of chronic pain in clinics [6]. In addition, it is difficult to explain that local injection of anesthetic procaine showed inhibition of the EA analgesic effect [7]. All findings above remain to be explained. A previous study showed that nociceptive response stimulated by complete Freund's adjuvant (CFA) can be reduced by treatment with EA with dilatational 100 Hz and 2 Hz alternating frequencies (2/100 Hz) on ST36 [8], which is related to the peripheral endogenous opioid system [9].

Mas-related G protein-coupled receptor C (MrgprC) is an atypical opioid receptor activated by opioids. The unique endogenous ligand of MrgprC is bovine adrenal medulla 22 peptide (BAM22), a degradation product of proenkephalin. BAM22 can be combined with *μ*, *δ*, and *κ* opioid receptors, but its activation on MrgprC cannot be antagonized by naloxone [10]. MrgprC is dramatically expressed in medium and small neurons of the DRG and trigeminal ganglion [11, 12], and then a section of MrgprC transports to lamina II of the spinal cord dorsal horn (SCDH) [11]. The highly restricted distribution of MrgprC makes it a potential therapeutic target with fewer central side effects [13]. Recent studies have shown that MrgprC plays an important role in modulating chronic inflammatory pain [10–12, 14] by inhibiting the increased expression of neuronal nitric oxide synthase (nNOS), calcitonin gene related peptide (CGRP), and c-fos in the DRG or SCDH [15]. Our previous studies have shown that MrgprC may be involved in the analgesic effect of EA [16], but the specific mechanism is unclear.

TRPV1 is an integration factor of nociceptive signals (heat, cold, and inflammation factors) in the DRG, and it is one of the key mechanisms that induce the thermal pain sensitization [17]. Both bilateral and unilateral EA at ST36 can inhibit the expression of TRPV1 in the DRG and SCDH under conditions of chronic inflammatory pain induced by CFA, which can also trigger reduction of a series of pain-sensitive molecules such as phosphorylated extracellular regulated protein kinases (p-ERK), phosphorylated cAMP-response element binding protein (p-CREB), phosphorylated P38 (p-p38), and others [18]. However, limited data have been obtained on regulatory effects of EA on TRPV1 in the spinal cord and how TRPV1 is regulated by EA in chronic inflammatory pain. TRPV1 has been demonstrated to be a major downstream target in MrgprC-activating signaling pathways in acute inflammatory pain [14, 19, 20], and it is mediated by PKC [21]. MrgprC might be involved in the modulation of pain sensitization through the PKC/TRPV1 pathway in the DRG and SCDH.

In this study, to observe MrgprC in the EA analgesic effect, we established chronic inflammatory pain by subcutaneously injecting CFA and blocking MrgprC expression by intrathecally administrating MrgprC small interfering RNA (siRNA). By applying intrathecal administration of bovine adrenal medulla 8-22 (BAM8-22), a C-terminal artificial hydrolytic fragment of BAM22, the MrgprC-specific agonist, as a control, we observed TRPV1 expression, TRPV1 translocation from the cytoplasm to the PM, and the level of p-TRPV1 mediated by PKC in the DRG and SCDH to investigate the possible mechanisms of MrgprC modulation of peripheral pain sensitization by EA.

## 2. Materials and Methods

### 2.1. Animals

Adult male Sprague-Dawley rats (280–300 g) were purchased from the BK (Shanghai Sippr-BK Laboratory Animal Co. Ltd., China) and housed individually at 25 ± 2°C under a 12 h alternating light-dark cycle with food and water ad libitum. The treatment of animals was strictly performed in accordance with the guidelines of the regulations of the State Science and Technology Commission for the Care and Use of Laboratory Animals (State Science and Technology Commission Number 2, 1988). All efforts were made to minimize animal suffering and to reduce the number of animals used in this study.

### 2.2. Intrathecal Catheter Implantation

As Yaksh and Rudy previously reported [22], rats were implanted with indwelling catheters into the lumbar vertebra subarachnoid. Briefly, a rat was anesthetized with 7.5% sodium chloral hydrate. A polyethylene catheter (PE, OD 0.50 mm × I.D 0.25 mm, RWD Life Science Co., Ltd., China) was inserted into the subarachnoid space from the back of L5-L6 lumbar intervertebral till its tip was positioned at the L4-L5 spinal cord. The other end of the catheter was anchored in the back of the lumbar. A successful indication was a temporary motor paralysis of the bilateral lower limbs induced by intrathecal lidocaine (0.1 g/5 mL; Otsuka Pharmaceutical Co., Ltd., China) 24 h after catheter implantation. The rats were allowed to recover for 5-6 days. Only rats without evidence of neurologic deficits were chosen for the experiment.

### 2.3. Establishment of Model and Experimental Groups

Inflammatory pain was induced by subcutaneously injecting (sc) 0.1 mL CFA (Sigma, St. Louis, USA) into the left hinder paw. The rats were divided randomly into 5 groups: normal group, model group, EA + control siRNA group, EA + MrgprC siRNA group, and BAM8-22 group (*n* = 8 per group). The normal group was injected with 0.1 mL saline, while the other 4 groups received CFA injection.

### 2.4. Treatments

As Ndong and his colleagues reported [14], MrgprC siRNA sequences 5′-CAUGUC AGCUAUUAUAUGUt-ps-t-3′ and 3′-t-ps-t GUACAGUCGAUAAUA UACA-5′ (GenePharma Co., Ltd., China) and mismatch control siRNA sequences 5′-CAAGUUAUCUAG UAAUUAUa-ps-t-3′ and 3′-t- ps-aGUUCAAUAGAUCAU UAAUA-5′ (GenePharma Co., Ltd., China) were used. In these sequences, “t” and “a” represented 2′-O-methyluridine and 2′-O-methyladenine, respectively, and “ps” was a phosphorothioate linkage. SiRNAs were dissolved in transfection regent i-Fect™ (Neuromics, USA) at a concentration of 5 *μ*g/*μ*L and were applied 1 d, 2 d, 3 d, 4 d, 5 d, and 6 d after CFA injection. BAM8-22 (10 nmol, 10 *μ*L, Abcam Co., Ltd., USA), a selective agonist of MrgprC, was administered intrathecally 2 d and 5 d after CFA injection.

As described previously [23], rats were immobilized loosely by cloth cover. Aseptic acupuncture needles (0.25 mm in diameter) were inserted into the bilateral Zusanli (ST36, 5 mm lateral to the anterior tubercule of the tibia) and Kunlun (BL60, at the ankle joint level and between the tip of the external malleolus and tendon calcaneus) since both acupoints were reported to be adept in treating inflammatory pain [24]. The terminals of unilateral couple needles in ST36 and BL60 were connected to electrodes from the HANS Acupuncture point nerve stimulator (LH-202H, Huawei Co., Ltd., China). No anesthesia was used during the EA treatment. The 2/100 Hz EA (alternation of 2 Hz and 100 Hz, 0.2 ms pulse width) was applied at an intensity of 1 mA for the first 15 min and 2 mA for another 15 min for a total of 30 min once per day in the EA + control siRNA and EA + MrgprC siRNA groups.

### 2.5. Measurement of Thermal Hyperalgesia

All tests were performed during the light phase. To observe thermal hyperalgesia, the paw withdrawal latency (PWL, s) was measured with a plantar test apparatus (37370, UGO Basile, Italy). Room temperature was maintained at 25 ± 2°C during the test. Rats were adapted in a plastic chamber (69 cm × 17 cm × 14 cm) on an elevated glass plate for 30 min. The radiant heat source was positioned under the center of the hind paw, and the withdrawal latency was recorded. The intensity of the light source was adjusted to obtain the PWL of normal animals within approximately 10 s to 15 s. A cut-off time of 20 s was set to protect the animal from tissue damage. PWLs were measured 1 d before CFA injection and 1 d, 2 d, 3 d, 4 d, 5 d, and 6 d after CFA injection (chronic inflammatory pain phase). At any test point, PWL was measured 3 times at 5-minute intervals and averaged values were calculated.

### 2.6. Total Protein and Cytoplasmic and Plasma Membrane (PM) Proteins Extraction

Ipsilateral DRG and SCDH from rats were harvested and homogenized in RIPA lysis buffer with added 1 mM PMSF and phosphatase inhibitor (Na3VO4 and NaF). The lysate was centrifuged at 14,000*g* for 5 min at 4°C and the supernatant was collected as total protein. Cytoplasmic and PM proteins were fractionated following the manufacturer's protocol of the Plasma Membrane Protein Extraction Kit (BioVision Inc., USA) [25]. To extract cytoplasmic protein, ipsilateral DRG and SCDH were lysed in Homogenization Buffer on ice by inserting and rotating for approximately 70–80 times until 70%~80% of the neurons were lysed under light microscopy. Then, the lysate was centrifuged at 700 ×g for 10 min at 4°C to remove nuclei and unlysed cells. Cytoplasmic protein was extracted from centrifuged supernatant (at 14,000*g* for 30 min at 4°C). The total membrane pellet was resuspended in the upper phase solution with 1 mM PMSF; then the lower phase solution was added, incubated on ice for 5 min, and centrifuged at 1000 ×g for 5 min at 4°C. The upper phase solution was carefully collected and then centrifuged at 15,000 ×g for 10 min at 4°C. Remove the supernatant; the pellet was the PM protein and can be dissolved in RIPA before further use. All proteins were then quantitated by BCA protein assay (Beyotime Institute of Biotechnology, Shanghai, China) and were stored at −80°C. All proteins were analyzed by immunoblotting.

### 2.7. Western Blot (WB)

20 *μ*g protein were separated by 8.0% SDS-polyacrylamide gel electrophoresis and transferred to a polyvinylidene fluoride (PVDF) membrane. After transfer, the PVDF membranes were blocked with 5% (w/v) skim milk in Tris-buffered saline (TBST) [10 mM Tris HCl (pH 7.5), 150 mM NaCl, and 0.1% Tween-20] for 1 h at room temperature (RT). The PVDF membranes were then blotted with rabbit polyclonal PKC*γ* antibody (1 : 2000, Abcam Co., Ltd., USA) or rabbit monoclonal PKC*ε* antibody (1 : 1000, Santa Cruz, USA), or rabbit TRPV1 polyclonal antibody (1 : 1000, Abcam Co., Ltd., USA), or rabbit p-TRPV1 polyclonal antibody (1 : 1000, Abcam Co., Ltd., USA), or rabbit monoclonal GAPDH antibody (1 : 10000, Abcam Co., Ltd., USA), or rabbit polyclonal Na^+^-K^+^-ATPase antibody (1 : 1000, Cell Signaling Technology, USA) for the DRG and SCDH samples at 4°C overnight in 5% skim milk. Then, the samples were incubated with HRP conjugated goat anti-rabbit antibody for 2 h at RT, the bands were detected by chemiluminescence using Western ECL kit (Abcam Co., Ltd., USA), and densitometry was performed by Image Quant LAS 4000 (GE, USA). The integrated optical density (IOD) of the bands was analyzed by Image Quant TL7.0 Analysis Software (GE, USA).

### 2.8. Immunofluorescence Assay

The rats were deeply anesthetized intraperitoneally with sodium chloral hydrate (0.35 mL/100 g) and then perfused with 4% paraformaldehyde in 0.1 M phosphate buffer (pH 7.4, 4°C). SCDH and DRG were fixed in 4% paraformaldehyde for 4 h, transferred into 15% sucrose overnight and then 30% sucrose for 48 h in 0.1 M phosphate buffer (pH 7.4) for cryoprotection, and stored at −80°C. DRG at 12 mm and SCDH at 30 mm were cut by freezing microtome (HM550, Thermo Fisher Scientific, USA) and mounted on slides. To permit comparisons across treatment groups, sections from different groups were processed simultaneously. The slides were incubated in 10% (w/v) normal goat serum for 1 hour and then incubated overnight at 4°C in rabbit polyclonal TRPV1 antibody (DRG, 1 : 1000; SCDH, 1 : 400, Abcam Co., Ltd., USA) or rabbit polyclonal PKC*ε* antibody (1 : 500, Abcam Co., Ltd., USA) or rabbit monoclonal PKC*γ* (1 : 500, Abcam Co., Ltd., USA). After washing, slides were transferred to anti-goat IgG H&L (Alexa Fluor @645) (TRPV1, 1 : 400; PKC*ε*, 1 : 200, Abcam Co., Ltd., USA) or anti-rabbit IgG H&L (Alexa Fluor @488) (PKC*γ*, 1 : 200, Abcam Co., Ltd., USA) for 1 hour at 37°C. Sections were mounted on glass slides, air-dried, and covered with coverslips by Aquamount (Thermo Fisher Scientific, USA). Images were captured at 100x magnification by a laser scanning confocal microscope (Nikon A1R/A1, Japan). Quantification of immunoreactivity (IR) was performed by calculating the number of TRPV1-IR neurons in the total number of neurons in the DRG and the total optical density of the positive neuron in laminae I-II of the SCDH in each section using Image-Pro Plus 6.0 software. Five sections per animal were randomly selected, and the mean ratio was determined. All DRG neurons were sectioned through their nuclei.

### 2.9. Statistical Analysis

All data are expressed as the mean ± SEM. Statistically significant differences among groups were determined by one-way analysis of variance (ANOVA). The differences for multiple comparisons were performed by least significant difference (LSD) testing (if there was variance homogeneity) and Dunnett's* T*3 test (if there was variance heterogeneity). *P* < 0.05 was considered statistically significant in this study.

## 3. Results

### 3.1. Intrathecal Administration of MrgprC siRNA Blocks Analgesic Effect of EA

**Figure 1:**
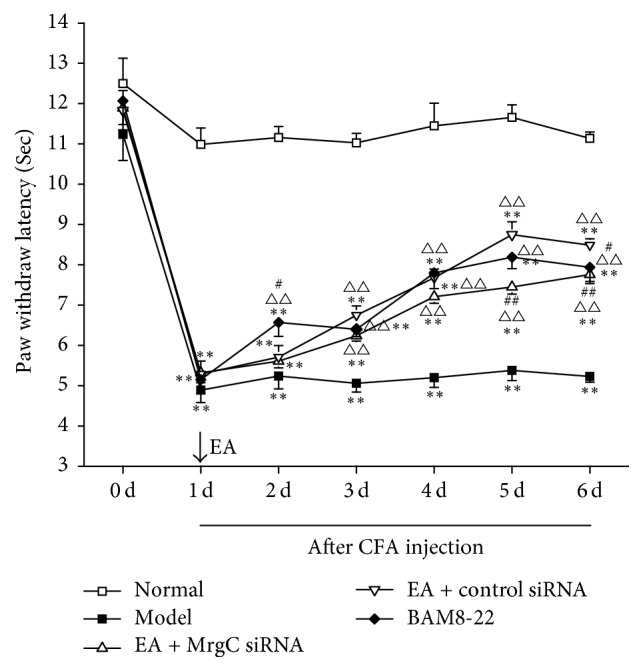
Behavioral test at different time point. Ipsilateral PWLs at different time points were assessed by plantar test apparatus. Data are expressed as the mean ± SEM (*n* = 8). ^*∗∗*^*P* < 0.01 compared to normal group, ^△△^*P* < 0.01, compared to model group, and ^##^*P* < 0.01 and ^#^*P* < 0.05 compared to EA + control siRNA group.

The ipsilateral PWL of rats was used to assess thermal hyperalgesia (Figure 1). Before CFA injection, there were no differences among groups. Compared with the normal group, PWLs in the group with CFA injection showed a drastic decrease on day 1 after CFA injection (*P* < 0.01). Compared with the model group, the PWLs of rats given EA or BAM8-22 increased gradually at each time point from day 3 or day 2 (*P* < 0.01, *P* < 0.01, resp.). There was a significant reduction in PWL in the EA + MrgprC siRNA group compared with the EA + control siRNA group when MrgprC siRNA was intrathecally administered on day 5 and day 6 (*P* < 0.01, *P* < 0.01, resp.).

### 3.2. Regulation of EA on p-TRPV1 and TRPV1 Translocation, but Not TRPV1 Expression, Is Inhibited by MrgprC siRNA in the DRG

**Figure 2:**
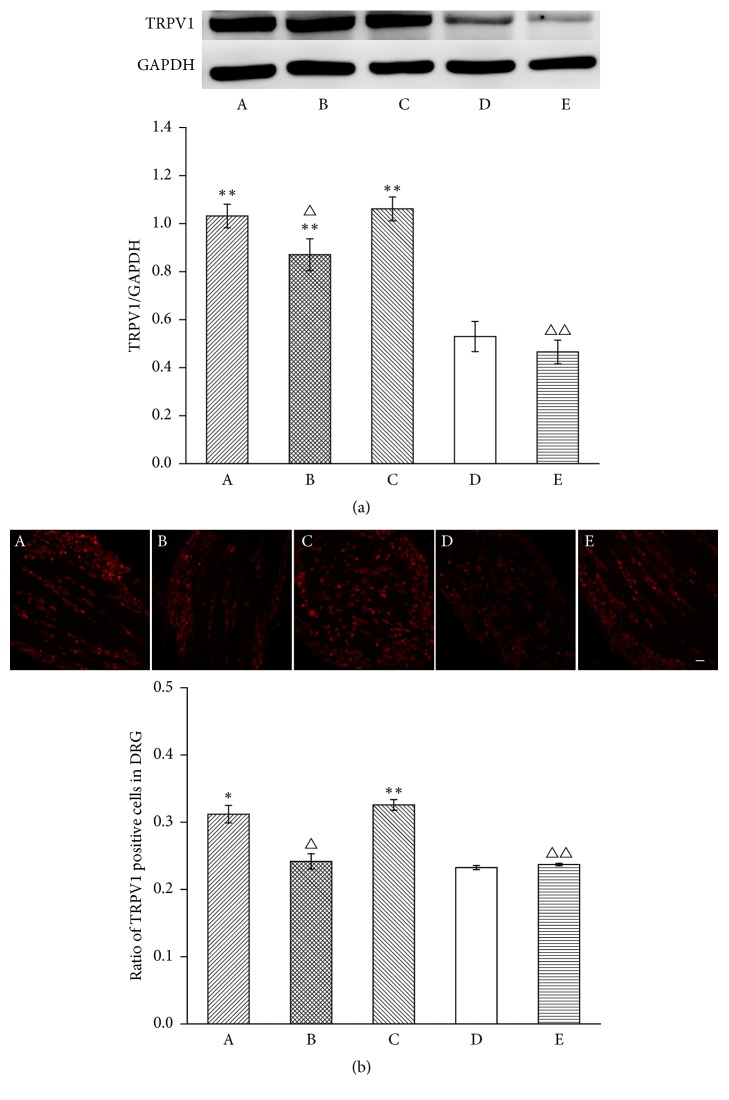
TRPV1 expression in ipsilateral DRG. (a) Results from WB are expressed as relative density of TRPV1 (GAPDH serves as the commonly used internal reference protein) (*n* = 4). (b) TRPV1 positive neurons were mainly expressed in small-to-medium DRG cells. The results from immunofluorescence are expressed as proportion of TRPV1-IR positive cells over whole stained cells (*n* = 4). Scale bar = 100 *μ*m. Data are expressed as the mean ± SEM. ^*∗*^*P* < 0.01 and ^*∗∗*^*P* < 0.01 compared to normal group; ^△^*P* < 0.05 and ^△△^*P* < 0.01 compared to model group. A: EA + MrgprC siRNA group, B: EA + control siRNA group, C: model group, D: normal group, and E: BAM8-22 group.

To investigate the influence of MrgprC on EA modulation of the TRPV1 pathway in the DRG, we observed the expression of TRPV1, TRPV1 translocation, and p-TRPV1. Compared with the normal group, TRPV1 expression in the DRG by WB markedly increased on the 6th day after CFA injection (*P* < 0.01). Six doses of EA downregulated the expression of TRPV1 compared with the model group (*P* < 0.05). However, intrathecal MrgprC siRNA had no effect on TRPV1 expression in the EA + MrgprC siRNA group compared with the EA + control siRNA group. Intrathecal BAM8-22 also significantly downregulated expression of TRPV1. Immunofluorescence was also performed to detect TRPV1 expression. Positive TRPV1-IR was highly distributed in small neurons. In line with data from WB (Figure 2(a)), immunofluorescence (Figure 2(b)) data showed CFA injection increased TRPV1 expression in the model group (*P* < 0.01). The increase was attenuated by EA (*P* < 0.05) or intrathecal BAM8-22 (*P* < 0.01). Intrathecal MrgprC siRNA did not inhibit the downregulation of TRPV1 by EA.

**Figure 3:**
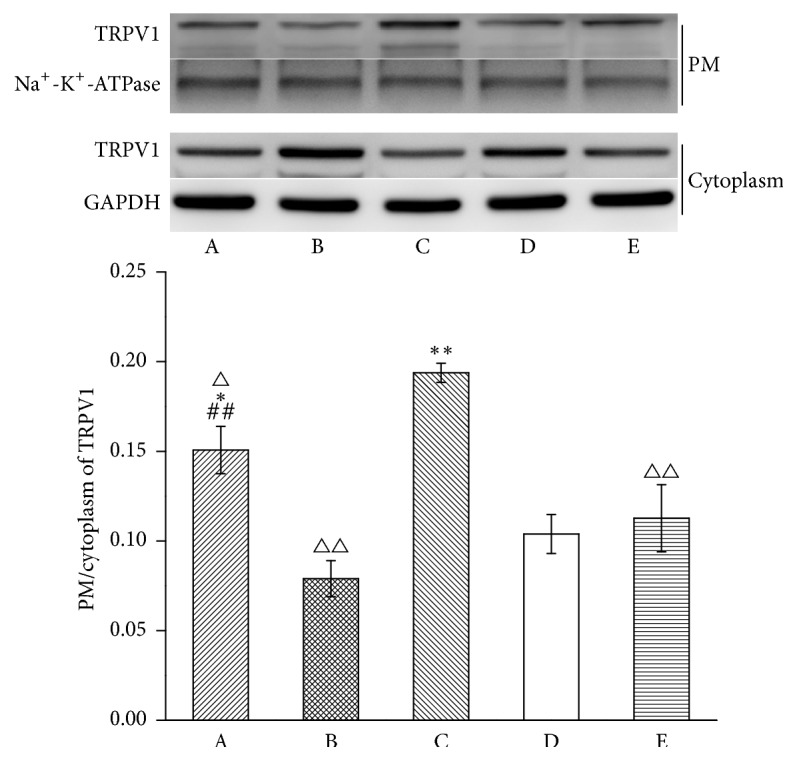
Translocation of TRPV1 in ipsilateral DRG. The results from WB are expressed as relative density of TRPV1 in the PM (Na^+^-K^+^-ATPase serves as the commonly used internal reference protein in the PM and GAPDH serves as the commonly used internal reference protein in the cytoplasm) (*n* = 4). Data are expressed as the mean ± SEM. ^*∗*^*P* < 0.05 and ^*∗∗*^*P* < 0.01 compared to normal group; ^△^*P* < 0.05 and ^△△^*P* < 0.01 compared to model group; ^##^*P* < 0.01 compared to EA + control siRNA group. A: EA + MrgprC siRNA group, B: EA + control siRNA group, C: model group, D: normal group, and E: BAM8-22 group.

To explore the influence of MrgprC siRNA on TRPV1 translocation, the expression of TRPV1 in the PM and the cytoplasm was detected (Figure 3). The translocation of TRPV1 obviously increased in the model group when compared with the normal group (*P* < 0.01). EA and intrathecal BAM8-22 both resulted in a decrease in translocation (*P* < 0.01, *P* < 0.01). Compared with EA + control siRNA group, intrathecal MrgprC siRNA can effectively suppress EA modulation on the translocation of TRPV1 (*P* < 0.01).

**Figure 4:**
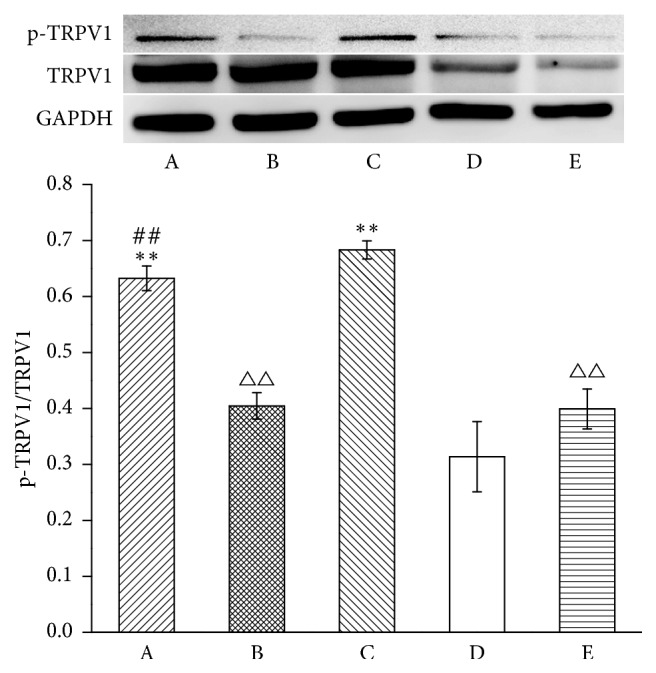
p-TRPV1 level in ipsilateral DRG. Results from WB are expressed as relative density of p-TRPV1 (equal loading was verified by assaying TRPV1) (*n* = 4). Data are expressed as the mean ± SEM. ^*∗∗*^*P* < 0.01 compared to normal group; ^△△^*P* < 0.01 compared to model group; ^##^*P* < 0.01 compared to EA + control siRNA group. A: EA + MrgprC siRNA group, B: EA + control siRNA group, C: model group, D: normal group, and E: BAM8-22 group.

Then, we observed the alteration of p-TRPV1 levels in the DRG (Figure 4). Levels of p-TRPV1 in the model group were higher than in the normal group (*P* < 0.01). EA downregulated p-TRPV1 levels in EA + control siRNA groups compared with the model group (*P* < 0.01). Intrathecal MrgprC siRNA obviously inhibited downregulation of EA on p-TRPV1 (*P* < 0.01). Compared with the model group, p-TRPV1 levels in the BAM8-22 group were significantly reduced in DRG (*P* < 0.01).

### 3.3. Regulation of EA on p-TRPV1 and TRPV1 Translocation, but Not TRPV1 Expression, Is Inhibited by MrgprC siRNA in the SCDH

**Figure 5:**
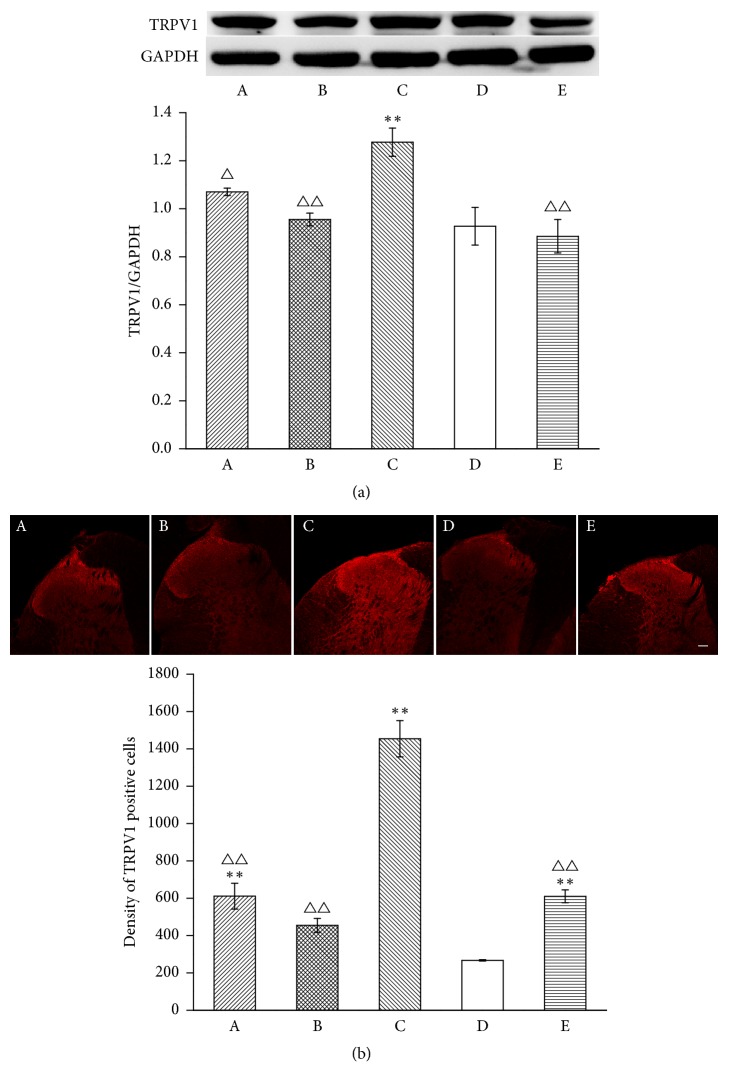
TRPV1 expression in ipsilateral SCDH. (a) Results from WB are expressed as relative density of TRPV1 (GAPDH serves as the commonly used internal reference protein) (*n* = 4). (b) TRPV1 positive neurons were mainly expressed in superficial layer of SCDH (laminae I-II). The results from immunofluorescence are expressed as total density of TRPV1-IR positive cells in laminae I-II (*n* = 4). Scale bar = 100 *μ*m. Data are expressed as the mean ± SEM. ^*∗∗*^*P* < 0.01 compared to normal group; ^△^*P* < 0.05 and ^△△^*P* < 0.01 compared to model group. A: EA + MrgprC siRNA group, B: EA + control siRNA group, C: model group, D: normal group, and E: BAM8-22 group.

We then explored the influence of MrgprC siRNA on expression of TRPV1, TRPV1 translocation, and p-TRPV1 in the SCDH through WB and immunofluorescence. The TRPV1 expression obviously increased in the model group when compared with the normal group (*P* < 0.01). EA and intrathecal BAM8-22 both resulted in a decrease in TRPV1 expression (*P* < 0.01, *P* < 0.01). Nevertheless, expression in the EA + MrgprC siRNA group was not significantly higher than that in the EA + control siRNA group when intrathecally administered with MrgprC siRNA (Figures 5(a) and 5(b)).

**Figure 6:**
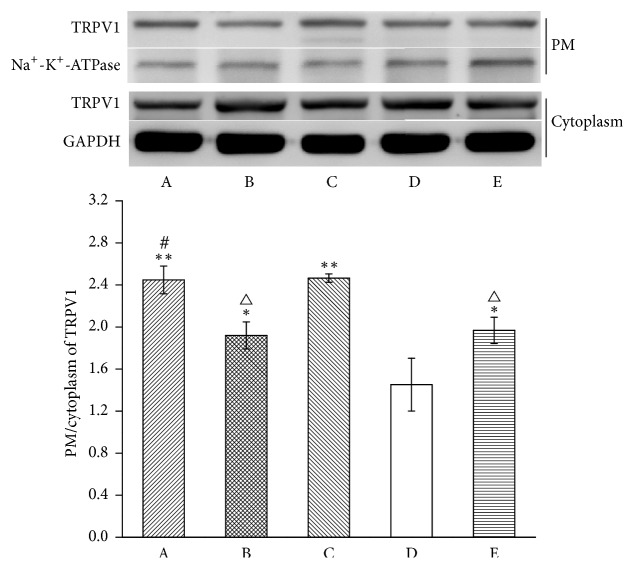
Translocation of TRPV1 in ipsilateral SCDH. The results from WB are expressed as relative density of TRPV1 in the PM (Na^+^-K^+^-ATPase serves as the commonly used internal reference protein in the PM and GAPDH serves as the commonly used internal reference protein in the cytoplasm) (*n* = 4). Data are expressed as the mean ± SEM. ^*∗*^*P* < 0.05 and ^*∗∗*^*P* < 0.01 compared to normal group; ^△^*P* < 0.05 compared to model group; ^#^*P* < 0.05, compared to EA + control siRNA group. A: EA + MrgprC siRNA group, B: EA + control siRNA group, C: model group, D: normal group, and E: BAM8-22 group.

The influence of MrgprC siRNA on the translocation of TRPV1 was next investigated (Figure 6). The translocation obviously increased in the model group when compared with the normal group (*P* < 0.01). EA and intrathecal BAM8-22 both resulted in a decrease in translocation (*P* < 0.05, *P* < 0.05). Translocation in the EA + MrgprC siRNA group was higher than in the EA + control siRNA group when intrathecally administered with MrgprC siRNA (*P* < 0.05).

**Figure 7:**
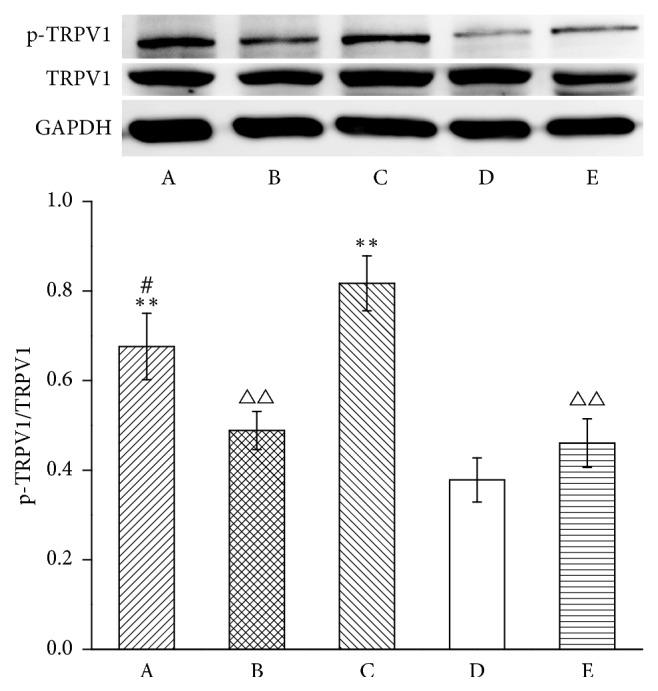
p-TRPV1 level in ipsilateral SCDH. Results from WB are expressed as relative density of p-TRPV1 (equal loading was verified by assaying TRPV1) (*n* = 4). Data are expressed as the mean ± SEM. ^*∗∗*^*P* < 0.01 compared to normal group; ^△△^*P* < 0.01 compared to model group; ^#^*P* < 0.05 compared to EA + control siRNA group. A: EA + MrgprC siRNA group, B: EA + control siRNA group, C: model group, D: normal group, and E: BAM8-22 group.

We also observed an alteration of p-TRPV1 levels in the SCDH (Figure 7). p-TRPV1 levels in the model group were higher than those in the normal group in the SCDH (*P* < 0.01). EA downregulated p-TRPV1 in the EA + control siRNA group compared with the model group (*P* < 0.01). However, intrathecal MrgprC siRNA obviously inhibited the effect of EA on p-TRPV1 (*P* < 0.05). Compared with the model group, the p-TRPV1 levels in the BAM8-22 group were significantly reduced (*P* < 0.01).

### 3.4. MrgprC siRNA Blocks Downregulation Effect of EA on PKC*ε* in the DRG but Not on PKC*γ* in the SCDH

**Figure 8:**
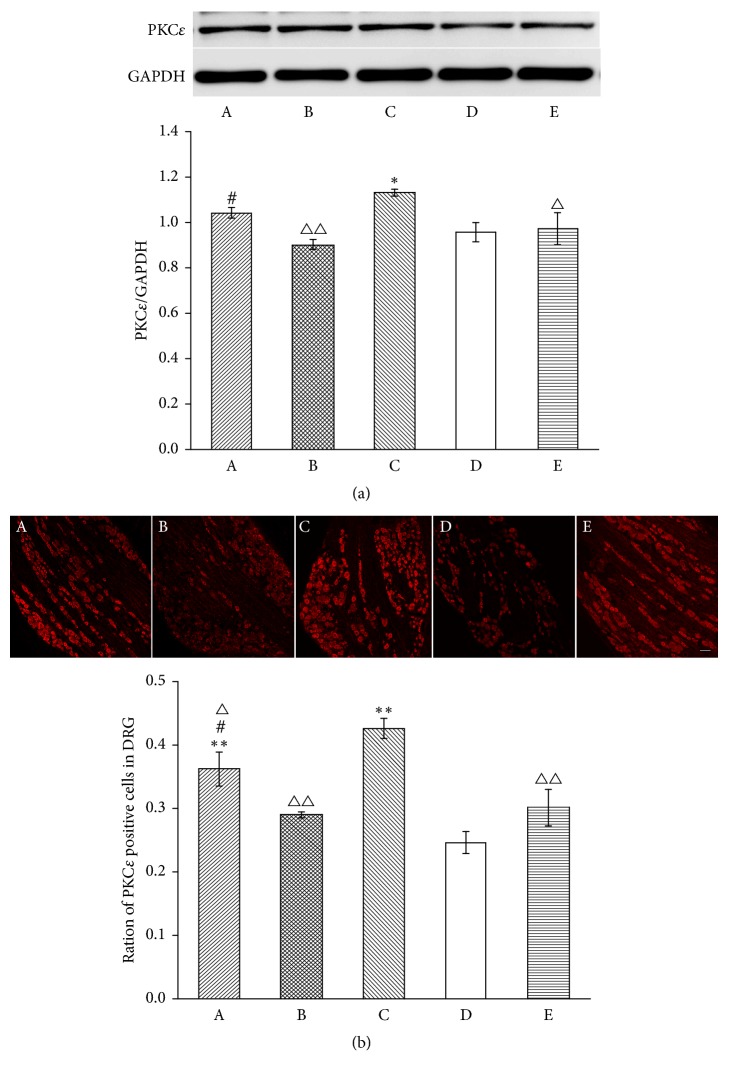
Expression of PKC*ε* in ipsilateral DRG. (a) Results from WB are expressed as relative density of PKC*ε* (GAPDH serves as the commonly used internal reference protein) (*n* = 4). (b) PKC*ε*-IR positive neurons were mainly expressed in small-to-medium DRG cells. The results from immunofluorescence are expressed as ratio of PKC*ε*-IR positive cells over whole stained cells (*n* = 4). Scale bar = 100 *μ*m. Data are expressed as the mean ± SEM. ^*∗*^*P* < 0.05 and ^*∗∗*^*P* < 0.01, compared to normal group; ^△^*P* < 0.05 and ^△△^*P* < 0.01 compared to model group; ^#^*P* < 0.05 compared to EA + control siRNA group. A: EA + MrgprC siRNA group, B: EA + control siRNA group, C: model group, D: normal group, and E: BAM8-22 group.

**Figure 9:**
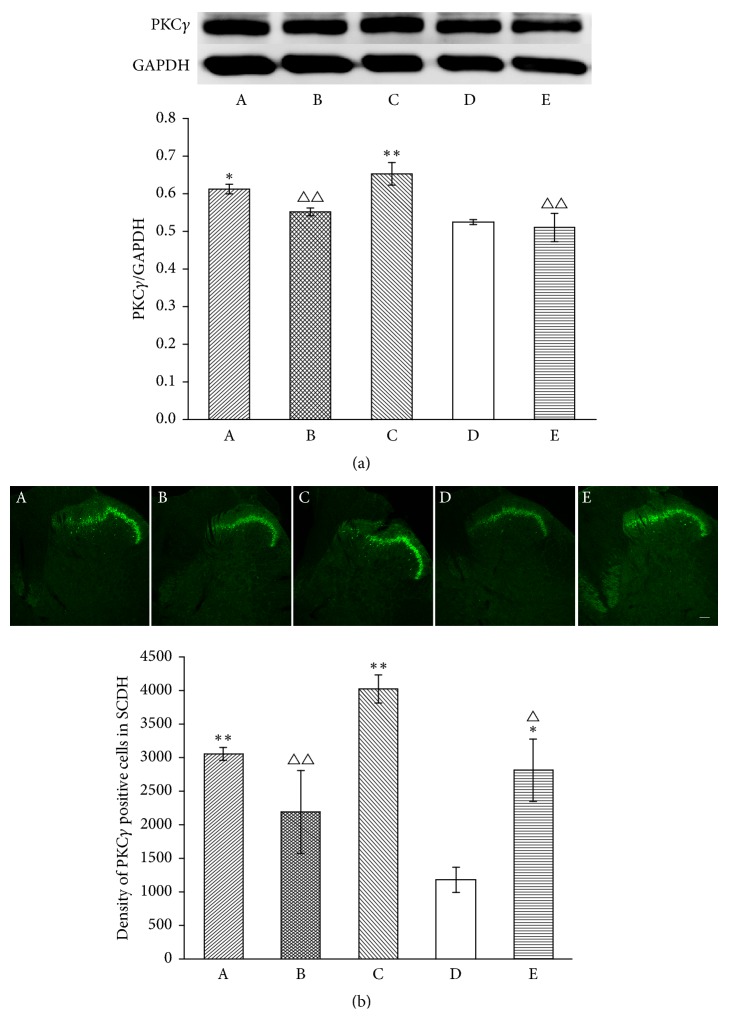
Expression of PKC*γ* in ipsilateral SCDH. (a) Results from WB are expressed as relative density of PKC*γ* (GAPDH serves as the commonly used internal reference protein) (*n* = 4). (b) PKC*γ*-IR positive neurons were mainly expressed in lamina II of superficial spinal dorsal horn. The results from immunofluorescence are expressed as density of PKC*γ*-IR positive cells in lamina II (*n* = 4). Scale bar = 100 *μ*m. Data are expressed as the mean ± SEM. ^*∗*^*P* < 0.05 and ^*∗∗*^*P* < 0.01 compared to normal group; ^△^*P* < 0.05 and ^△△^*P* < 0.01 compared to model group. A: EA + MrgprC siRNA group, B: EA + control siRNA group, C: model group, D: normal group, and E: BAM8-22 group.

We next investigated whether MrgprC modulated the EA intervention through PKC in the DRG and SCDH as it is linked closely to the phosphorylation of TRPV1 [26]. PKC*ε* is one of the key subtypes of PKC highly expressed in the DRG, and PKC*γ* is highly expressed in the SCDH. WB and immunofluorescence were performed to investigate these expressions (Figures 8 and 9). The data from WB showed that the CFA injection produced an increase in PKC*ε* (*P* < 0.05) and PKC*γ* (*P* < 0.01) in the model group compared to the normal group on the inflamed side. The increase was significantly inhibited by EA (PKC*ε*, *P* < 0.01; PKC*γ*, *P* < 0.01). When MrgprC siRNA was intrathecally administered, expression of PKC*ε* was significantly higher in the EA + MrgprC siRNA group than in the EA + control siRNA group, but there was no difference in PKC*γ* levels between them. Meanwhile, intrathecal BAM8-22 attenuated CFA-induced increase in PKC*ε* (*P* < 0.05) in the DRG and PKC*γ* in the SCDH (*P* < 0.01).

Immunofluorescence was also used to observe the expression of PKC. The data from immunofluorescence were consistent with the data from WB. Figures 8(b) and 9(b) showed that there were basal levels of PKC*ε* in the DRG and PKC*γ* in the SCDH. Compared with the normal group, CFA injection caused a robust increase in PKC*ε* (*P* < 0.01) in the DRG and PKC*γ* (*P* < 0.01) in the SCDH. EA downregulated the expression of PKC*ε* (*P* < 0.01) and PKC*γ* (*P* < 0.01) compared with the normal group. When MrgprC siRNA was intrathecally administered, the regulation effect of EA was blocked in the DRG but not in the SCDH. In addition, the increase in PKC*ε* (*P* < 0.01) and PKC*γ* (*P* < 0.05) induced by CFA was also inhibited by BAM8-22.

## 4. Discussion

Peripheral sensitization includes thermal hyperalgesia and mechanical allodynia, which are the main features of chronic inflammatory pain. Ipsilateral hyperalgesia is generated within 4 h after CFA modeling and may last for 4–6 weeks, while contralateral hyperalgesia generally occurs 18 days after CFA modeling [27]. Our previous study showed that there was no effect on mechanical allodynia with intrathecal administration of MrgprC siRNA that was sufficient for blocking MrgprC expression in the DRG [28]. Therefore, ipsilateral PWL was observed to assess thermal hyperalgesia in this study. After subcutaneously injecting with CFA for 1 day, the ipsilateral hind paws of rats showed redness, swelling, and heat. The PWL of the model group was significantly lower than that of the saline group from 1 d to 6 d, which suggested that the model was successfully established. Previous studies demonstrated that 2/100 Hz EA stimulating bilateral ST36 and the BL60 can alleviate thermal hyperalgesia induced by chronic inflammation [8, 16, 23], especially on the 5th and 9th day after CFA modeling [29]. We observed 6 instances of EA alleviating thermal hyperalgesia in this study.

MrgprC is one of the members of the superfamily associated with CGRPs, and its gene is highly expressed in the pain-related peripheral nervous system (DRG, trigeminal ganglion) that is closely related to pain transmission. The Mrg receptor family is classified as MrgprA–MrgprH in rodents [11] and MrgprX1–MrgprX7 in humans [30]. A latest research shows MRGPRX is a promising therapeutic target for treating persistent pain because of its unique expression in nociceptors within the peripheral nervous system [31]. Studies have shown that rodent MrgprC and human MrgprX are structurally highly homologous [32]. Therefore, elucidating the role of MrgprC in pain can contribute to a better understanding of the role of human MrgprX in pain, since there was no direct evidence that MrgprC had a modulatory effect on chronic inflammatory pain as an MrgprC-specific inhibitor had not been found yet. The current studies are limited to investigating the effect of different MrgprC agonists on pain and effects of MrgprC in the early stage of CFA models (24–48 h) [33, 34]. These studies showed that intrathecal discontinuous injection of BAM8-22 could suppress thermal hyperalgesia in chronic inflammatory pain. Consistent with other papers, our early research showed that intrathecal injection of MrgprC siRNA more than 5 times can block 50–60% of MrgprC gene expression [14, 28, 35]. To elucidate the influence of MrgprC on EA analgesia, MrgprC siRNA was intrathecally injected 6 times with mismatched siRNA as a control. The results showed that the analgesic effect of EA decreased after applying MrgprC siRNA. Considering our previous data [16, 36] showed that MrgprC expression in the DRG but not in the SCDH and BAM22 release in both the DRG and the SCDH could be increased by EA in CFA-induced chronic inflammatory pain, the results indicated that MrgprC may play a role in modulation of EA over a 6-day period of chronic inflammatory pain, and the possible analgesic mechanism of EA is associated with its upregulation on MrgprC in the DRG and activation of MrgprC by BAM22 in both the DRG and SCDH.

Persistent inflammatory injury can trigger and maintain nociceptive thermal hyperalgesia, and TRPV1 is usually considered to play a key role in inflammatory and thermal pain [37]. TRPV1 is highly expressed in neurons in the DRG [38], and deletion of TRPV1 results in thermal hyperalgesia elicited by CFA [39]. Consistent with previous research, TRPV1 expression was significantly increased in the DRG and SCDH 6 days after CFA modeling in this study. The results of the immunofluorescence assay indicated that the TRPV1 increase was mainly in DRG small neurons and superficial layer of the SCDH. In the 28-day chronic pain caused by CFA, TRPV1 in isolectin B4 (IB4) positive (signal of MrgprC positive DRG neurons [40]) DRG neurons was consistently overexpressed in the first 14 days and further transported into the superficial layer of the SCDH [41]. TRPV1 was shown to be a major downstream target in the MrgprC-induced cell signaling pathway in acute inflammatory pain, and the effect of MrgprC on TRPV1 was mediated by PKC [21]. It is presumed that MrgprC may suppress CFA-induced hyperalgesia at the DRG and SCDH levels based on these data. Upon expression, a part of MrgprC is transported to the terminal of primary afferent synapses in superficial layer II of the SCDH that is bound by PKC*γ* [42]. MrgprC activated by BAM22 can downregulate the expression of CGRP in the L3–L5 layer of the SCDH and DRG and inhibit occurrence and development of inflammatory hyperalgesia induced by CFA in the early stage (24 h) [43]. Intrathecal injection of BAM8-22 attenuated CFA-induced thermal hyperalgesia and inhibited ERK phosphorylation in the DRG and at the cytomembrane PKC*γ* level in the SCDH [3]. Thus, we observed how MrgprC takes part in EA analgesia through TRPV1 pathways in the DRG and SCDH. The present results showed EA and BAM8-22 effectively inhibited TRPV1 expression, whereas MrgprC did not participate in EA inhibition of TRPV1 expression in the DRG and SCDH.

At the subcellular level, TRPV1 mostly seems to be a kind of sequestered intracellular compartments [44]. In the process of inflammatory pain, TRPV1 can be activated by SP, calcitonin peptide, and others [45]; then, it translocates to the PM from the cytoplasm and is phosphorylated by PKC/PKA and finally the function of TRPV1 is activated [46]. In this study, we observed the expression of TRPV1 in the PM and cytoplasm to investigate the translocation of TRPV1 in the DRG and SCDH. The results showed that the translocation of TRPV1 was increased in chronic inflammatory pain and the suppression of EA could be reversed by MrgprC siRNA. It has been confirmed that MrgprC-contained primary afferent fibers terminate in the middle region of the SCDH that is rich in PKC*γ* subtype neurons [33, 40, 47]. Therefore, the function of MrgprC might be closely related to PKC*γ*. A previous study showed that inhibition of the phosphorylation of PKC*ε* at the ser729 site in DRG promoted by MrgprC could inhibit the functional activation of PKC*ε* [28]. In this experiment, it was observed that both EA and BAM8-22 could inhibit the upregulation of inflammation on expression of PKC and PKC-mediated phosphorylation of TRPV1 in the DRG and SCDH. Furthermore, MrgprC siRNA reversed the inhibition of EA on the expression of PKC*ε* and p-TRPV1 but did not inhibit the expression of PKC*γ* in the SCDH, which suggested other potential ways for EA modulation on MrgprC to decrease the phosphorylation of TRPV1 in the SCDH. MrgprC was involved in the regulation of EA on the translocation and the phosphorylation of TRPV1 via PKC in different ways in the DRG and SCDH.

In this study, it was observed that PKC*ε* in the DRG, PKC*γ* in the SCDH, the translocation, and phosphorylation of TRPV1 in the DRG and SCDH were significantly increased after 6 days of CFA modeling, the upregulation of which was inhibited by BAM8-22. Thus, MrgprC may be involved in the modulation of chronic inflammatory pain through the PKC/TRPV1 pathway in the DRG and SCDH. EA also inhibited the translocation and phosphorylation of TRPV1 as well as the expression of PKC*ε* in the DRG and PKC*γ* in the SCDH. MrgprC siRNA reversed the effect of EA on PKC*ε* expression, the translocation, and the phosphorylation of TRPV1 but did not reverse PKC*γ* expression, which suggested another potential way for EA modulation on MrgprC to decrease the phosphorylation of TRPV1 in the SCDH. All the above data showed that MrgprC was involved in EA regulation of the PKC/TRPV1 pathway in different ways in the DRG and SCDH.

## 5. Conclusions

In summary, this current study shows a novel finding that EA and MrgprC both play a role in chronic inflammatory pain regulation by inhibiting expression, translocation, and PKC-mediated phosphorylation of TRPV1 in the DRG and SCDH. MrgprC may be involved in EA analgesia through the PKC/TRPV1 pathway in the DRG and SCDH in different ways. The above results demonstrate the functional role of EA in pain signaling and provide a novel peripheral mechanism for involvement of MrgprC, an atypical opioid receptor, in EA regulation of chronic inflammatory pain.
